# The Evolving Role of Ivabradine for Focal Atrial Tachycardia: A Systematic Review

**DOI:** 10.1002/joa3.70312

**Published:** 2026-03-10

**Authors:** Haikal Balweel, Rifqi Rizkani Eri, Sania Zahrani, Kiara Hanna Quinncilla, Novaro Adeneur Tafriend, Agus Harsoyo

**Affiliations:** ^1^ Gatot Soebroto Army Central Hospital Jakarta Indonesia; ^2^ National Cardiovascular Center Harapan Kita Jakarta Indonesia; ^3^ Bagas Waras General District Hospital Klaten Central Java Indonesia; ^4^ Faculty of Medicine Universitas Indonesia Jakarta Indonesia

**Keywords:** arrhythmia, focal atrial tachycardia, ivabradine

## Abstract

**Background:**

Ivabradine was initially introduced for heart failure and coronary artery disease patients, with its interesting feature of heart rate reduction without reducing heart contractility or blood pressure. In recent years, however, the use of ivabradine evolved for arrhythmia, including for focal atrial tachycardia (FAT). This systematic review aims to evaluate all the existing evidence on ivabradine for focal atrial tachycardia and synthesize information on its use, including efficacy and safety.

**Methods:**

A comprehensive literature search was conducted in three large databases: PubMed, ScienceDirect, and Scopus, following the PRISMA guidelines, using the search terms of: (ivabradine) AND (atrial tachycardia). Studies were included if they reported the use of Ivabradine for FAT in humans. Studies on atrial tachycardia outside of focal mechanisms or any other arrhythmias were excluded.

**Results:**

The search strategy resulted in 375 articles: 63 from PubMed, 262 from ScienceDirect, and 50 from Scopus, which subsequently resulted in 19 articles included for final review after a meticulous filtering process. The included studies were 11 case reports, 4 case series, and 4 observational studies discussing the outcome of ivabradine for focal atrial tachycardia.

**Conclusion:**

Evidence from small cohorts and case reports suggests promising outcomes and a good safety profile of ivabradine for FAT, but larger, well‐designed and more robust studies are needed. Recognizing an automaticity‐driven FAT is key to considering ivabradine, especially when FAT persists despite cardioversion or standard therapy.

## Graphical abstract

Overview of the evolving role of ivabradine in focal atrial tachycardia (FAT), highlighting its HCN‐mediated mechanism, features of ivabradine‐sensitive FAT, hypothesized predictors of response, special populations requiring special considerations, and potential adverse effects.

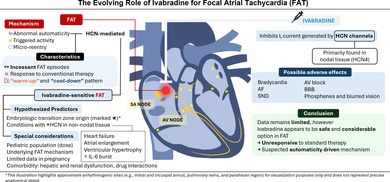

## 1 Introduction

The sinoatrial (SA) node is the heart's natural pacemaker, firing at 60–100 beats per minute [1]. In atrial tachycardia (AT), however, ectopic atrial foci override the SA node and may discharge at a significantly higher rate [2]. This may lead to devastating outcomes, acutely as lethal tachyarrhythmia, acute heart failure, or even mortality, and chronically as tachycardia‐induced cardiomyopathy (TICM) [2, 3]. The underlying mechanisms are thought to vary, including abnormal automaticity, often referred to as focal AT (FAT), and electrical reentry, including micro‐ and macroreentry [4, 5, 6]. This mechanistic heterogeneity poses challenges in determining optimal treatment strategies, particularly for focal AT, which is often incessant and shows limited response to antiarrhythmic drugs, electrical cardioversion, or even catheter ablation [6, 7].

One promising emerging therapeutic option is ivabradine, a relatively novel drug that selectively inhibits the I_f_ current generated by hyperpolarization‐activated cyclic nucleotide‐gated (HCN) channels responsible for pacemaker activity in the SA node, originally developed for stable angina and heart failure. The BEAUTIFUL [8], SHIFT [9], and SIGNIFY [10] trials established its ability to reduce heart rate without affecting contractility or blood pressure and confirmed its overall tolerability. In recent years, beyond its sinoatrial nodal effects, emerging evidence suggests that ivabradine may also suppress ectopic atrial activity [11]. This has generated increasing interest, particularly for focal AT cases presenting with hemodynamic instability or left ventricular dysfunction, in which conventional therapies, such as beta‐blockers, calcium channel blockers, class I–III antiarrhythmics, cardioversion, or catheter ablation may be ineffective or unsuitable [2, 12].

**FIGURE 1:**
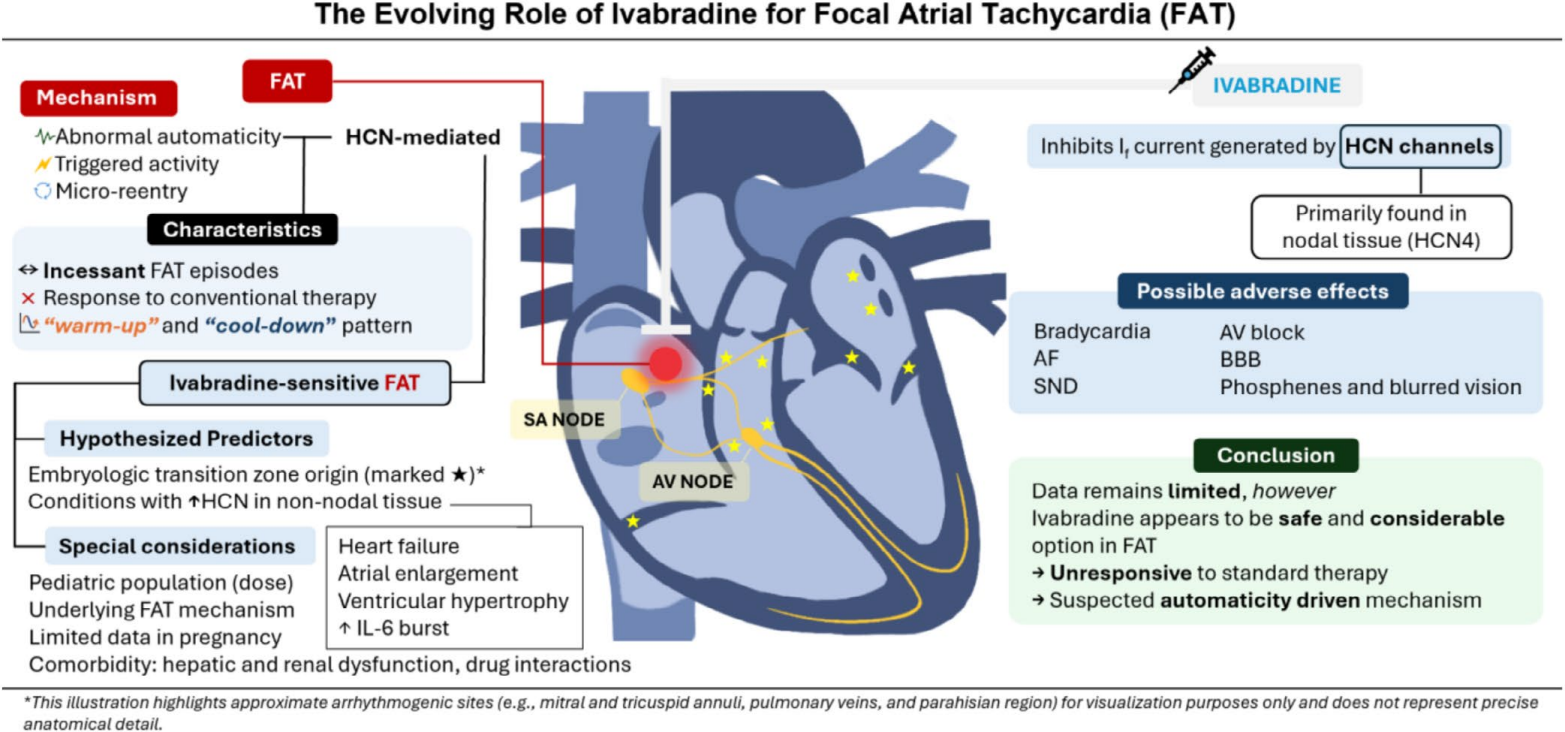
The evolving role of ivabradine for focal atrial tachycardia (FAT).

This systematic review synthesizes current evidence on ivabradine for focal AT, examines its electrophysiological basis, and identifies gaps for future research (Figure 1).

## 2 Methods

### 2.1 Study Selection

This systematic review was conducted in reference to the Preferred Reporting Items for Systematic Reviews and Meta‐Analyses (PRISMA) guidelines. From inception to October 24, 2025, we conducted a comprehensive search throughout three databases: PubMed, Scopus, and ScienceDirect, using the following term: (ivabradine) AND (atrial tachycardia) (Table S1). Studies were included if they report the use of Ivabradine for AT in any form between case report, case series, observational studies, cohort studies or randomized controlled‐trials (RCT) in human. Studies are excluded if they investigate the use of Ivabradine in any other type of arrhythmia besides atrial tachycardia, or any other atrial tachycardia beside focal mechanism. Language restrictions were applied, leading to full‐papers not written in English being excluded. The study selection process was conducted by 4 independent authors (H.B., R.R.E, S.Z., K.H.Q.).

### 2.2 Data Extraction and Study Quality Assessment

Following title, abstract and full‐text screening, included articles were retrieved for data extraction and quality assessment. Data extraction and study quality assessment were independently performed by four authors (H.B., R.R.E, S.Z., and K.H.Q.) using a pre‐established standardized sheet. The authors extracted the following informations: author, year of publication, title of the article, article type, clinical characteristics, dosing and administration strategy of Ivabradine, clinical outcomes after Ivabradine administration (including arrhythmia termination, heart rate reduction, symptom improvement, hemodynamic stabilization, and adverse events, if applicable), and mechanistic insight of Ivabradine for AT (if applicable).

For the quality assessment, Joanna Brigghs Institute (JBI) Critical Appraisal Checklist for Case Reports [13] was utilized to assess case reports and series, while Newcastle‐Ottawa Scale (NOS) [14] was used for non‐randomized studies, and Cochrane Risk of Bias Tool 2 (RoB 2) [15] was used for randomized studies. JBI checklist was utilized by evaluating eight domains: patient demographics, clinical history and timeline, presenting clinical condition, diagnostic assessment, interventions, post‐intervention outcomes, adverse or unanticipated events, and takeaway lessons. Meanwhile, NOS was used by assessing these components: study group selection, comparability, and ascertainment of outcome of interest. Additionally, Cochrane RoB 2 tool was utilized by assessing these five domains: bias arising from the randomization process, bias due to deviations from intended interventions, bias due to missing outcome data, bias in measurement of the outcome, bias in selection of the reported result. The result of the study selection, data extraction, and study quality assessment were resolved through discussion with all co‐authors (H.B., R.R.E., S.Z., K.H.Q., N.A.T., and A.H.).

## 3 Results

**FIGURE 2:**
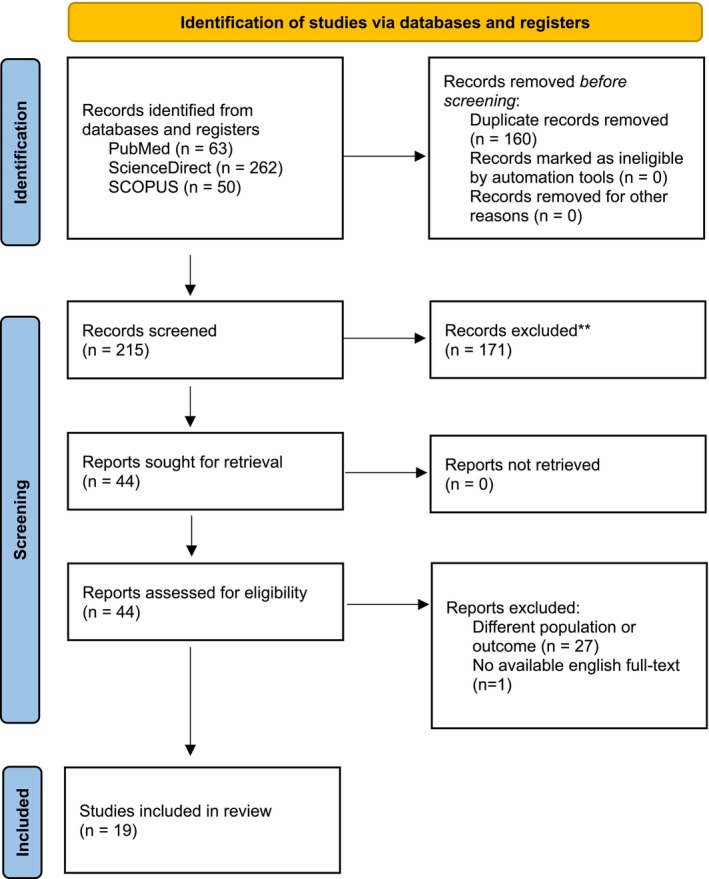
PRISMA flow chart of search strategy.

The initial search found 375 articles: 63 from PubMed, 262 from ScienceDirect, and 50 from Scopus. After removing 160 duplicates, 215 articles remained. Following title and abstract screening, 171 articles were excluded due to irrelevance, leaving 44 for full‐text review. Nineteen articles were then excluded: 18 for addressing unrelated topics or being in review format, and one because the full text was not available in English. After meticulous review for the full‐paper, an additional 6 articles were excluded due to different population and intervention in concept. In total, 19 articles were included in the final review (Figure 2) [16, 17, 18, 19, 20, 21, 22, 23, 24, 25, 26, 27, 28, 29, 30, 31, 32, 33, 34].

A total of 19 studies were included in this review, consisting of 15 case reports or series and 4 observational studies. No randomized controlled trials were identified. The majority of the available evidence comes from case‐based literature and small cohorts, highlighting the early stage of clinical experience with ivabradine in atrial tachycardia.

### 3.1 Study and Baseline Characteristics

Nineteen studies were included, comprising four cohort studies, twelve single‐patient case reports, and three small case series. Ten studies focused exclusively on pediatric patients, most of whom were infants or young children presenting with incessant or drug‐refractory focal atrial tachycardia (FAT), often in the context of congenital heart disease or post‐cardiac surgery [16, 20, 23, 24, 25, 26, 27, 30, 31, 34]. Five studies reported adult‐only populations, typically young adults with symptomatic focal AT, with some cases complicated by tachycardia‐induced cardiomyopathy [19, 22, 29, 31, 33]. Four studies included both adult and pediatric patients [17, 21, 28, 31].

Tachycardia‐induced cardiomyopathy (TIC) was present in several reports, with markedly reduced LVEF documented in critically ill pediatric patients [26, 27]. In adults with refractory arrhythmia, TIC was also reported [21, 22, 31, 33]. Other cases reported preserved LV function despite persistent arrhythmia [23, 24, 25, 29, 30].

Most patients had previously received multiple antiarrhythmic agents, including beta‐blockers, amiodarone, flecainide, sotalol, or digoxin, as well as adenosine or electrical cardioversion, with limited efficacy. In this context, ivabradine was introduced either as a primary therapeutic strategy for rate and rhythm control [16, 23, 24, 26, 29]. It was used as an adjunct in critically ill or refractory cases [20, 27, 30]. Ivabradine also served as a bridging therapy to ablation [18, 19, 31, 33]. Some patients continued ivabradine after declining ablation [17, 22].

Notably, one case involved a pregnant woman with incessant FAT beginning in the third trimester [28]. Ivabradine at 5 mg twice daily achieved arrhythmia control and stabilization of LV function, with spontaneous resolution 1 month postpartum and delivery of a healthy infant at term. Baseline characteristics of all included studies are summarized in Table 1.

**TABLE 1:** Baseline characteristics of the included studies.

No.	First Author (Year)	Study design	No. of patients included	Population	Sex	Age	TIC	LV function	Prior anti‐arrhythmic therapy	Ivabradine strategy
*Cohort studies included*										
1.	Tolani (2023) [16]	Prospective cohort	15	Pediatric with congenital heart disease	Female 40%	Mean of 7 months (1–18)	N/A	N/A	Digoxin, flecainide, and propranolol	As primary strategy for rate and rhythm control (33%) or as second‐ or first‐line strategy (67%)
2.	Hai‐Yang (2022) [17]	Prospective cohort	3	Adult, Pediatric	N/A	Mean of 37.8 ± 19.4 years	N/A	N/A	N/A	Failure or refusal to undergo ablation
3.	Xu (2022) [18]	Retrospective cohort	12	Pediatric	Female 50.0%	Mean of 7.5 ± 4.5 years	50.0%	Mean LVEF 53.0% ± 18.9%	Beta‐blocker, amiodarone, propafenone, and sotalol	As bridging strategy to ablation; or continued if refusing ablation
4.	Banavalikar (2019) [19]	Prospective cohort	28	Adult	Female 60.7%	Mean of 34.6 ± 21.5 years	25.0%	Mean LVEF 54.7% ± 14.3%	Beta‐blocker (metoprolol and propranolol) and amiodarone	As bridging strategy to ablation; or continued if refusing ablation
*Case reports included*										
5.	Balweel (2025) [20]	Case report	1	Post TOF repair pediatric, post‐cardiac arrest	N/A	1‐year	No	Normal LV function	Amiodarone, bisoprolol, propranolol, electrical cardioversion	As an additional therapy for rate and rhythm control in a critically ill patient, and stopped once SR is achieved
6.	Aggarwal (2024) [21]	Case series	1	Adult	F	22 year	Yes	LVEF 20%	N/A	Unsuccessful ablation
7.	Ahmed (2023) [22]	Case report	1	Adult	M	19 years	Yes	LVEF 20%–25%	Beta‐blocker	Refusal to undergo ablation
8.	Gul (2023) [23]	Case report	1	Pediatric	M	11 month	No	Normal LV function	Propranolol, amiodarone, sotalol, flecainide, digoxin	As primary strategy for rate and rhythm control
9.	Penslar (2023) [24]	Case report	1	Pediatric (Newborn) with ASD, VSD, PDA, Bicuspid PV	F	0 days	N/A	N/A	Flecainide (administered maternally during pregnancy) and propranolol (after birth)	As primary strategy for rate and rhythm control
10.	Jadhav (2023) [25]	Case report	1	Pediatric	F	7 years	No	Normal LV function	Metoprolol	As an additional therapy for rate and rhythm control, then stopped if possible
11.	Tasci (2022) [26]	Case report	1	Pediatric	M	15‐day	Yes	Reduced LV function	Amiodarone IV, oral propranolol, electrical cardioversion	As primary strategy for rate and rhythm control
12.	Karmegaraj (2021) [27]	Case series	Case 1	Pediatric, haemodynamically unstable, post‐cardiac arrest	M	60 days	Yes	LVEF 17%	Adenosine, metoprolol, digoxin, amiodarone, sotalol, electrical cardioversion	For hemodynamic stability restoration in a critically ill patient; and later as strategy to maintain SR
			Case 2	Pediatric, haemodynamically unstable	M	30 days	Yes	LVEF 19%	Adenosine	For hemodynamic stability restoration in a critically ill patient; and later as strategy to maintain SR
13.	Tonko (2021) [28]	Case report	1	Pregnant Woman; FAT onset at 3rd trimester	F	38 years	Yes	LVEF 46%	Maximal dose of bisoprolol	As primary strategy for rate control; later stopped when postpartum
14.	Das (2021) [29]	Case report	1	Adult	M	23 years	No	Normal LV function	Metoprolol	As primary strategy for rate and rhythm control in patients with failed ablation
15.	Michel (2020) [30]	Case report	1	Pediatric	F	12 month	No	Normal LV function	Adenosine, flecainide, amiodarone, and electrical cardioversion	For hemodynamic stability restoration in a critically ill patient; and later as primary strategy to maintain SR
16.	Janson (2019) [31]	Case series	Case 1	Pediatric	F	11 years	No	Normal LV function	Esmolol	As primary strategy for rate and rhythm control
			Case 2	Adult	M	26 years	N/A	N/A	Propranolol	As primary strategy for rate and rhythm control
			Case 3	Pediatric, post‐transplant	F	14 years	N/A	N/A	Nadolol and flecainide	As primary strategy for rate and rhythm control
17.	Meles (2015) [32]	Case report	1	Adult	F	18 years	Yes	LVEF 40%	Verapamil, flecainide, hypotension, amiodarone, atenolol, electrical cardioversion	As bridging strategy to ablation
18.	Al‐Musaad (2014) [33]	Case series	Case 1	Adult	M	27 years	Yes	LVEF 25%	Beta‐blockers and amiodarone	As primary strategy for rate and rhythm control, later as a bridging strategy to ablation
			Case 2	Adult	F	24 years	N/A	N/A	None, first‐line option was ivabradine	
19.	Bohora (2010) [34]	Case series	1	Pediatric	F	15	Yes	LVEF 40%	Beta‐blockers, amiodarone, and digoxin	As bridging strategy to ablation

### 3.2 Ivabradine Dosing

Dosing regimens varied. In adults, fixed oral dosing of 5 mg twice daily was most common [4, 19, 29, 31, 33]. Lower starting doses such as 2.5 mg twice daily were also used [22]. One cohort instead applied a weight‐based regimen of 0.28 mg/kg/day in two divided doses [18]. In pediatric populations, most studies used 0.05–0.1 mg/kg twice daily, with titration up to 0.2–0.3 mg/kg/day when needed [16, 17, 20, 23, 24, 25]. Lower ranges were also reported, such as 0.024–0.05 mg/kg BID [24, 30]. and 0.025 mg/kg increasing to 0.05 mg/kg/day [24]. Higher doses up to 0.15–0.2 mg/kg BID were reported in infants [23, 27]. One study also administered intravenous ivabradine in a pediatric cohort [18]. Ivabradine dosing of all included studies is summarized in Table 2.

**TABLE 2:** Ivabradine administration.

No.	First Author (Year)	How to administer ivabradine	When to stop ivabradine administration	Therapeutic target	ISAT	Time to AT termination	Ablation procedure	Follow‐up duration (median)	Follow‐up outcome	Adverse effect reported
*Cohorts included*										
1.	Tolani (2023) [16]	Oral, 0.05 mg/kg/day Monotherapy (26%) or as combination (74%) with flecainide, amiodarone, beta‐blockers, digoxin, or dexmedetomidine.	If adverse events arise (usually bradycardia); the ivabradine dose was withheld or halved, or replaced with other antiarrhythmic agent.	Sinus rhythm with termination of tachycardia within 24 h of ivabradine initiation	80%	< 24 h	No	N/A	N/A	Bradycardia in 47% of patients.
2.	Hai‐Yang (2022) [27]	Oral, 5 mg twice a day for adults; or 0.14 mg/kg/day divided into two doses in pediatric ≤ 50 kg	N/A	Sinus rhythm with normal HR	100%	N/A	Yes, unsuccessful	1.7 ± 0.6 years	N/A	None reported, well‐tolerated
3.	Xu (2022) [18]	Oral, 0.1 mg/kg every 12 h, increased to 0.2 mg/kg every 12 h if no restoration of stable sinus rhythm was observed after two doses	If neither rhythm nor heart rate control was observed within 48 h	Assessed using 24‐h Holter monitoring: stable sinus rhythm was restored (tachycardia burden was reduced to < 1%, success); the heart rate was reduced to an age‐acceptable level without slowing atrioventricular conduction (*partial success*), or the tachy‐ cardia burden was reduced to < 10% (*partial success*)	50.0% (25.0% success, and 25.0% partial success)	N/A	41.7%	5.0 months	FAT recurrence in 1 patient (8.3%)	None reported, well‐tolerated
4.	Banavalikar (2019) [19]	Oral, 0.28 mg/kg twice a day in children; 20 mg twice a day in adults. It is recommended to give a trial of ivabradine (10 mg single dose)	If non‐responsive within 12 h of ivabradine initiation	Termination of tachycardia with sinus rhythm (complete success) or HR < 100 bpm without termination (partial success)	64.0% (60.7% complete success, 2.3% partial success)	4.26 ± 0.53 h	75.0%	5.3 ± 3.2 months	No recurrence of FAT	None reported, well‐tolerated
*Case reports included*										
5.	Balweel (2025) [20]	Oral, 0.05 mg/kg twice a day As combination with amiodarone and digoxin	After SR is achieved	Sinus rhythm with normal HR	Yes	8 h	No	5 days (during hospitalization)	No recurrence of FAT	None reported, well‐tolerated
6.	Aggarwal (2024) [21]	N/A	N/A	Sinus rhythm with normal HR	Yes	N/A	Yes, unsuccessful	~3 years	No recurrence of FAT until the patient stopped the medication on his own; hence FAT resumes	N/A
7.	Ahmed (2023) [22]	Oral, 2.5 mg twice dail in addition to ongoing heart failure medication, including beta‐blocker	N/A	Sinus rhythm with normal HR	Yes	N/A	No	1 month	No recurrence of FAT and improvement of LVEF	None reported, well‐tolerated
8.	Gul (2023) [23]	Oral, single dose of 0.15 mg/kg and continued by 0.1 mg/kg/day twice a day	N/A	Sinus rhythm with normal HR	Yes	2 h	No	N/A	No recurrence of FAT	None reported, well‐tolerated
9.	Penslar (2023) [24]	Oral, 0.025 mg/kg twice daily, up‐titrated to 0.15 mg/kg twice daily	If free of FAT after the first year of life, ivabradine can be weaned off	Sinus rhythm with HR improvement	Yes	N/A	No	6 months	No recurrence of FAT before or after surgical closure of cardiac shunts	None reported, well‐tolerated
10.	Jadhav (2023) [25]	Oral, 0.025 mg/kg, up‐titrated to 0.5 mg/kg	If beta‐blocker is effective in pertaining rhythm control	Sinus rhythm with normal HR	Yes	N/A	No	N/A	No recurrence of FAT	None reported, well‐tolerated
11.	Tasci (2022) [26]	Oral, 0.05 mg/kg twice a day In combination with amiodarone when hospitalized; and with flecainide when discharged	N/A	Sinus rhythm with normal HR	Yes	1 h	No	7 days (during hospitalization)	One‐time recurrence of SVT after 12‐h of ivabradine administration, with an improvement of LV function	None reported, well‐tolerated
12.	Karmegaraj (2021) [27] Case 1	Enteral, 0.15 mg/kg In combination with digoxin and propranolol (when critically ill) In combination with propranolol (when discharged)	N/A	If haemodynamic‐ally stable, with restoration of sinus rhythm and normal HR	Yes	45 min	No	3 months	Haemodynami‐cally stable after ivabradine administration Short episodes of FAT but predominantly SR in 7‐d Holter monitoring and improvement of LVEF (70%)	None reported, well‐tolerated
	Case 2	Enteral, 0.15 mg/kg In combination with digoxin	N/A	If haemodynamic‐ally stable, with restoration of sinus rhythm and normal HR	Yes	4 h	No	5 days (during hospitalization)	Haemodynami‐cally stable after ivabradine administration, but symptom relief was supported by milrinone and furosemide	None reported, well‐tolerated
13.	Tonko (2021) [28]	Oral, 5 mg twice a day; up‐titrated to 7.5 mg twice a day	N/A	HR of ~100 bpm; can be stopped postpartum if SR is spontaneously restored	Yes	~6 h	No	Until 1 month postpartum	Spontaneously restored SR 1 month post‐partum; ivabradine was stopped	None reported, well‐tolerated
14.	Das (2021) [29]	Oral, 5 mg twice a day	N/A	Sinus rhythm with normal HR in 7‐day Holter monitoring with symptom relieve	Yes	N/A	Yes, unsuccessful	3 months	No recurrence of FAT and quite asymptomatic	None reported, well‐tolerated
15.	Michel (2020) [30]	Oral, 0.25 mg (0.025 mg/kg) every 12 h and increased to 0.5 mg (0.05 mg/kg) per dose on the second day. Combined with oral metoprolol (1 mg/kg/d).	N/A	Sinus rhythm with normal HR	Yes	2 days	No	2 and 10 weeks	Alternating ectopic atrial and sinus rhythm with a physiologic diurnal heart rate spectrum and a regular response to physical activity	None reported, well‐tolerated
16.	Janson (2019) [31] Case 1	Oral, 0.025 mg/kg twice a day	N/A	Sinus rhythm free of symptoms	Yes	90 min	No	6 months	No recurrence of FAT and symptom‐free	None reported, well‐tolerated
	Case 2	Oral, 2.5 mg twice a day	N/A	Free of symptoms	Yes	N/A	No	4 months	Symptom‐free	None reported, well‐tolerated
	Case 3	Oral, 2.5 mg twice a day, up‐titrated to 5 mg in the AM and 2.5 mg in the PM	When prolonged FAT resumed	Sinus rhythm	No	N/A	Yes, unsuccessful	2 months	After 2 months on ivabradine, a prolonged episode of FAT occurred; ivabradine was discontinued	None reported during ivabradine therapy, well‐tolerated
17.	Meles (2015) [32]	Oral, 5 mg twice a day	N/A	Sinus rhythm with normal HR	Yes	5 h	Yes	2 days of ivabradine, and then ablated	No recurrence of FAT with improvement of LVEF	None reported, well‐tolerated
18.	Al‐Musaad (2014) [33] Case 1	Oral, 7.5 mg twice a day	N/A	HR control	Yes	N/A	Yes	2.5 years	No recurrence of FAT with improvement of LVEF (55%) until the patient stopped the medication on his own	None reported, well‐tolerated
	Case 2	Oral, 7.5 mg twice a day With combination of metoprolol	When pregnant	HR control	Yes	N/A	Yes	> 1 years	No recurrence of FAT until the patient stopped the medication due to pregnancy	None reported, well‐tolerated
19.	Bohora (2010) [34]	Oral, 2.5 mg twice a day	N/A	HR < 100 bpm with symptom relieve	Yes	~2 days	Yes	2 months of ivabradine, and then ablated	No recurrence of FAT with improvement of LVEF (60%) until the patient stopped the medication on her own; hence FAT resumes	None reported, well‐tolerated

### 3.3 Anatomical Origin of Focal AT

Among the 19 studies included, seven provided information regarding the anatomical origin of focal atrial tachycardia (AT) identified during electrophysiological study (Table 3). Most of these cases originated from the right atrium, particularly the right atrial appendage, which appeared in several reports as the dominant site of ivabradine‐sensitive foci [17, 21, 31, 33]. In the prospective study by Hai‐Yang [17], two‐thirds of foci were located in the distal atrial appendage, while one‐third arose proximally, suggesting a potential gradient of automaticity within the appendage.

**TABLE 3:** Identification of FAT origin in patients undergoing EPS.

No.	First Auhor (Year)	Study design	No. of patients included	Location
1.	Michel (2020) [30]	Case report	1	Right atrial appendage
2.	Aggarwal (2024) [21]	Case series	1	The tip of the right atrial appendage
3.	Bohora (2010) [34]	Case report	1	The left superior pulmonary and LA appendage
4.	Meles (2015) [32]	Case report	1	Inferior wall of the common ostium of left pulmonary veins
5.	Das (2021) [29]	Case report	1	Mid cristal atrial tachycardia
6.	Al‐Musaad (2014) [33]	Case series	2	High right atrium below right atrial appendage; and right atrial appendage
7.	Hai‐Yang (2022) [17]	Prospective cohort	3	Proximal atrial appendage (33.3%) and distal atrial appendage (66.7%)

Other less common origins included the left atrial structures, such as the left superior pulmonary vein and the left atrial appendage [34]. The inferior wall of the common ostium of the left pulmonary veins was reported in one adult patient [31]. Additionally, one case described a focus in the mid‐crista terminalis [29]. Another identified the high right atrium just below the appendage [33]. Overall, these findings indicate that ivabradine‐responsive focal AT tends to arise from atrial regions with known pacemaker or automatic properties, particularly the atrial appendages. This recurring distribution supports the hypothesis that sites outside the sinus node may harbor If current activity susceptible to ivabradine inhibition.

### 3.4 Clinical Outcomes and Key Observations

Across nearly all reports, ivabradine was associated with a reduction in heart rate and termination of atrial tachycardia, often with rapid onset after initiation. Conversion to sinus rhythm was observed within hours in several cases: 2 h [30], 5 h [31], and 8 h [20], while others required longer, such as 24 h [26] or 72 h [27]. Sustained benefit was documented during follow‐up, including maintenance of sinus rhythm at 2 weeks [24] and freedom from recurrence at 3 months [29].

Recovery of left ventricular function was also a consistent finding in patients with tachycardia‐induced cardiomyopathy. Examples included improvement of LVEF from 40% to 60% after 1 month [34], and more rapid recovery within 24 h [26] or 72 h [27]. In the unique case of a pregnant woman with incessant focal AT, ivabradine stabilized ventricular function, enabled continuation of pregnancy, and was followed by spontaneous resolution of the arrhythmia 1 month postpartum [28].

Adverse events were uncommon, with bradycardia being the most frequent, generally mild and dose‐related [16]. Beyond clinical outcomes, some reports highlighted recurring anatomical patterns: ivabradine‐sensitive foci were often located in the atrial appendage, suggesting potential site‐specific susceptibility. Efficacy was consistently demonstrated in both pediatric and adult populations, reinforcing ivabradine's role as a promising therapeutic option in drug‐refractory or incessant focal AT. Clinical outcomes and key observations are summarized in Table 4.

**TABLE 4:** Key outcomes and notable findings of included studies.

No.	First Author (Year)	Key outcome(s)	Notable findings
1.	Tolani (2023) [16]	HR ↓, AT termination	Successful in 80% patients, bradycardia in 47% patients
2.	Hai‐Yang (2022) [17]	HR ↓, AT termination	No complication in 1.7 ± 0.6 years of follow up
3.	Xu (2022) [18]	HR ↓, AT termination	Well tolerated in patients with ↓ LVEF
4.	Banavalikar (2019) [19]	HR ↓, AT termination	Atrial appendage AT origin as a predictor of ivabradine response
5.	Balweel (2025) [20]	HR ↓, AT termination	AT terminated 8 h after ivabradine initiation following lack of response to beta‐blockers
6.	Aggarwal (2024) [21]	HR ↓, AT termination	Successful AT termination with Ivabradine after failed catheter ablation
7.	Ahmed (2023) [22]	HR ↓, AT termination	Improved LVEF in 1 month
8.	Gul (2023) [23]	HR ↓, AT termination	AT terminated 2 h after ivabradine initiation
9.	Penslar (2023) [24]	HR ↓, AT termination	Weaning off ivabradine therapy after 1 year
10.	Jadhav (2023) [25]	HR ↓, AT termination	Maintained sinus rhythm and symptoms‐free after 2 weeks
11.	Tasci (2022) [26]	HR ↓, AT termination	Recovered left ventricular function 24 h after ivabradine initiation
12.	Karmegaraj (2021) [27]	HR ↓, AT termination	Significantly improved LVEF 72 h after ivabradine initiation
13.	Tonko (2021) [28]	Spontaneous resolution of AT one month post‐partum	Improved LVEF, delivery of a healthy child at term, no growth retardation
14.	Das (2021) [29]	HR ↓, AT termination	No AT recurrence in 3 months, no adverse events
15.	Michel (2020) [30]	HR ↓, AT termination	Ivabradine combined with beta‐blocker
16.	Janson (2019) [31]	HR ↓, AT termination	No adverse events, symptoms‐free after 6 months
17.	Meles (2015) [32]	HR ↓, AT termination	AT termination 5 h after ivabradine initiation
18.	Al‐Musaad (2014) [33]	HR ↓, AT termination	Successful reversal of atrial‐tachycardia induced cardiomyopathy
19.	Bohora (2010) [34]	HR ↓, AT termination, reversal of tachycardia‐induced cardiomyopathy	Improved LVEF 1 month after ivabradine initiation

## 4 Discussion

Focal atrial tachycardia (FAT) often challenges conventional pharmacologic therapy. Beta‐blockers, calcium channel blockers, and even amiodarone may fail to control the rhythm, especially when the mechanism involves abnormal automaticity rather than reentry. Because FAT is often incessant, electrical cardioversion frequently proves ineffective or offers only transient success. The clinical efficacy of ivabradine in such cases therefore raises a compelling question: *how can a sinus node inhibitor suppress an atrial focus outside the node?*

Ivabradine targets the funny current (I_f_), an inward sodium–potassium current generated by hyperpolarization‐activated cyclic nucleotide‐gated (HCN) channels [11]. The activity of these channels is mainly modulated by intracellular cAMP and forms the molecular basis of spontaneous diastolic depolarization in pacemaker cells. Upon membrane repolarization, HCN channels open, allowing a slow inward flow that gradually brings the cell back toward threshold. Sympathetic stimulation enhances this process through cyclic AMP binding, which shifts channel activation to more positive potentials and accelerates firing [12]. Among the four HCN isoforms (HCN1–4), HCN4 predominates in the heart, especially within the sinoatrial node and other specialized conduction tissues. HCN channels are also present in ventricular myocytes, with HCN2 representing the predominant isoform. However, in the healthy adult ventricular myocardium, HCN expression is markedly lower than in the cardiac conduction system, rendering If currents largely undetectable under normal physiological conditions [11, 35]. During cardiac development, HCN channels are highly expressed in the embryonic ventricular myocardium; however, their expression diminishes progressively after birth and becomes largely confined to the specialized conduction system in healthy adult hearts [36].

Ivabradine inhibits HCN channels in an open‐state‐dependent manner by binding from the intracellular portion of the pore, with a predominant affinity to HCN4 compared to other HCN subtypes, slowing the slope of diastolic depolarization. The result is a slower spontaneous firing rate without affecting contractility, conduction velocity, or repolarization. Ivabradine's effect is intrinsically rate‐dependent: the faster the heart rate, the more frequently HCN channels open, and the more effective the drug becomes. This property inhibits hyperactive pacemaker tissue without affecting the rest of the myocardium. Clinically, ivabradine achieves pure heart‐rate reduction without negative inotropy or vasodilation, advantages in patients with impaired ventricular function or hemodynamic instability [11, 37].

### 4.1 Linking Ivabradine Electrophysiological Mechanisms to Focal AT

Focal AT may arise from enhanced automaticity, triggered activity, or microreentry [38]. The subset that responds dramatically to ivabradine, called ivabradine‐sensitive focal AT (ISAT), reflects an automatic mechanism driven by *I*_*f*_ [19]. In these foci, ectopic atrial cells appear to possess nodal‐like electrophysiological properties, expressing functional HCN channels that behave like miniature sinus nodes. By inhibiting these channels, ivabradine directly suppresses the abnormal pacemaker current driving the tachycardia [16]. Clinical observations support this concept. In reports by Janson et al. and Bohora et al., tachycardia recurred promptly when patients stopped ivabradine, confirming that continued HCN blockade was essential for rhythm stability [28, 31]. Conversely, ivabradine shows little effect in FAT due to microreentry, which relies on conduction heterogeneity rather than automatic firing [22].

The origin of HCN activity in ectopic atrial tissue remains speculative. In the pediatric population, one hypothesis suggests embryologic remnants of sinus venosus tissue that failed to fully “atrialize,” leaving clusters of pacemaker‐like cells in areas such as the atrial appendage or pulmonary veins, sites repeatedly identified as ivabradine‐sensitive [16]. Histologic studies offer mixed evidence: Matsuyama et al. [39] found nodal tissue near an ectopic focus at the inferior mitral annulus, whereas Hai‐Yang et al. [17] found none in excised atrial appendages, suggesting ordinary atrial myocytes may express *I*_*f*_ channels under certain conditions. Another possibility is that different HCN isoforms (e.g., HCN1‐3) contribute to localized automaticity [32].

HCN channels, particularly HCN2 and HCN4, are re‐expressed in ventricular hypertrophy heart failure, and inflammatoric conditions [40]. Overespression of HCN1 is also reported in human left atrial dilation [41]. The resulting increase in I_f_ current in ventricular myocytes may reduce repolarization reserve and prolong action potential duration, thereby increasing susceptibility to afterdepolarizations, and may also promote abnormal automaticity, collectively contributing to clinically significant ventricular arrhythmias [36, 42, 43, 44, 45]. Kuwabara et al. [46] reported that Ivabradine dramatically reduced number of PVCs, VT episodes, and prolongs survival in HCN overexpression induced cardiomyopathy mice models without significantly reducing heart rate or compromising cardiac function. They also reported that increased expression of HCN2 promotes susceptibility to arrhythmia in chronic β‐adrenergic stimulated conditions [46].

The recurring observation suggests that ivabradine is most effective when the arrhythmia's driver is automatic and HCN‐dependent [11]. This explains its consistent efficacy in incessant focal AT, inappropriate sinus tachycardia, and junctional ectopic tachycardia, arrhythmias linked by their dependence on the same pacemaker current. In short, ivabradine is effective in these arrhythmias because it directly suppresses the hyperactive pacemaker current that drives the tachycardia.

### 4.2 Ivabradine Dosage, Onset of Work, and Clinical Applicability for Focal AT

There is no universally agreed‐upon dose of ivabradine for focal atrial tachycardia (FAT). In adults, the doses reported in the literature ranged from 2 × 2.5 to 2 × 10 mg daily [16, 17, 18, 19, 20, 21, 22, 23, 24, 25, 26, 27, 28, 29, 30, 31, 32, 33, 34]. Some studies started with the lower end, 2 × 2.5 mg [22, 31], while others titrated up to 2 × 5 mg [17, 29, 32, 33], 2 × 7.5 mg [33], or 2 × 10 mg [19]. One report described the use of ivabradine in a pregnant woman, starting at 2 × 5 mg and later increased to 2 × 7.5 mg daily [28]. These doses align with current recommendations in the 2021 ESC guideline of Ivabradine for heart failure, which suggest initiating therapy at 2 × 5 mg and uptitrating to 2 × 7.5 mg [47]. In the pediatric population, dosing was calculated per body weight, ranging from 2 × 0.025 mg/kg to 2 × 0.2 mg/kg. Lower doses (2 × 0.025–0.05 mg/kg) were most common, presented in six reports [16, 24, 25, 26, 30, 31], while two studies used higher doses up to 0.2 mg/kg [18, 23]. Based on existing evidence, we presented a proposed clinical ivabradine initiation protocol on Table 5. Ivabradine was used as monotherapy in several cases [18, 23, 24, 25, 31, 34], while other reports combined it with beta‐blockers, amiodarone, flecainide, or digoxin (Table 6) [16, 20, 26, 27, 30].

**TABLE 5:** Proposed clinical ivabradine initiation protocol.

Patient group	Initiation dose^a^	Dose increment	Maximum dose^b^
Adult	2.5^a^ to 5 mg BID	Per 2.5 mg	10 mg BID
Pregnant women	5 mg BID	Per 2.5 mg	7.5 mg BID
Children	0.025 mg/kg BID	Per 0.025 to 0.05 mg/kg	0.2 mg/kg BID

^a^ Lowest dose reported.

^b^ Highest dose reported.

**TABLE 6:** Reported combined drugs to ivabradine.

Patient group	Drugs combined
Adult	Beta‐blockers; heart failure regiment
Pregnant women	N/A^a^
Children	Beta‐blockers; amiodarone; flecainide; digoxin

^a^ Only one paper reported ivabradine usage in pregnancy and used monotherapy approach.

The drug was administered in a variety of clinical scenarios. In some studies, it was the primary strategy for rhythm or rate control [16, 24, 26, 30, 31]. In others, it served as a bridge to ablation, as an alternative when ablation was declined or unsuccessful, or as add‐on therapy when prior treatments failed [17, 18, 19, 20, 21, 22, 23, 24, 25, 26, 27, 28, 29, 33, 34]. Meanwhile, in the other remaining studies ivabradine was used as an add‐on or second/third line therapy or as temporary rate or rhythm control strategy, in which if the rhythm was converted to sinus rhythm and HR target was achieved, ivabradine was discontinued [20, 21, 22, 23, 25, 27, 28, 32]. Time to FAT termination varied: in adults, it ranged from 4 to 6 h [19, 28, 32], while in children, it could occur anywhere from 45 min to 48 h [16, 20, 23, 26, 27, 30, 31, 34]. In comparison to current 2019 ESC guideline of SVT, ivabradine is used in inappropriate sinus tachycardia (IST) as a first‐line agent and in chronic therapy of FAT if the first‐line agents failed [48].

Three pediatric cases of critically ill, hemodynamically unstable FAT highlight ivabradine's potential [20, 27, 30]. All had failed conventional therapy, including adenosine, amiodarone, beta‐blockers, and electrical cardioversion, and two had even experienced cardiac arrest. Ivabradine stabilized all three patients, though the time to effect varied: as quickly as 45 min in combination with metoprolol and amiodarone, 8 h in another case, and about 2 days in a third case with esmolol infusion [20, 27, 30]. These cases highlight the importance of early recognition of automaticity‐driven FAT, as indicated by non‐responsiveness to adenosine, allowing timely administration of ivabradine. However, currently, the 2019 ESC guideline does not yet incorporate ivabradine for acute FAT therapy [48].

### 4.3 Considerations in Special Populations

#### 4.3.1 Pediatric Population

FAT in infants often resolves spontaneously, making medical therapy the preferred initial approach [23]. Catheter ablation may be required in incessant FAT or when left ventricular dysfunction is present, but its procedural risks are higher in low‐weight infants [23]. Meanwhile, currently, Ivabradine is not a first‐line antiarrhythmic in children and is primarily approved for heart rate reduction in heart failure, with beta‐blockers, such as sotalol or esmolol, remaining the first‐line therapy, either alone or combined with sodium‐channel blockers like flecainide [23, 26]. However, ivabradine may be considered as add‐on therapy in pediatric FAT when tachycardia is automatic and refractory to first‐line agents. One report recommended its use alongside beta‐blockers, which are generally well‐tolerated in children [16, 20, 23, 26, 27, 30, 31, 34]. This strategy is particularly useful when ablation is difficult due to patient size or when families decline invasive treatment.

#### 4.3.2 Pregnancy

Data on ivabradine use in pregnancy remain limited. One case report described treatment from the third trimester until 1 month postpartum with no adverse effects on either mother or child; the drug was discontinued once sinus rhythm returned after delivery [28]. Another case delayed ivabradine initiation until after pregnancy termination [33]. In highly symptomatic patients, pharmacotherapy may be considered as a bridge to delivery, although current guidelines advise against ivabradine use in pregnant or breastfeeding women [26, 39]. German Embryotox data suggest no major teratogenic risk, but the evidence base remains insufficient [48, 49, 50]. Similarly, in clinical trials of ivabradine in heart failure (SHIFT) [10] and coronary artery disease (BEAUTIFUL and SIGNIFY) [8, 10], several women became pregnant; among those with follow‐up data, growth restriction occurred in two cases and premature birth in one, but no fetal abnormalities were reported. Regulatory authorities remain cautious, with the FDA recommending its use only in the third trimester when benefits outweigh risks, while the British National Formulary advises against ivabradine during pregnancy.

#### 4.3.3 Early Postoperative FAT in Congenital Heart Disease (CHD)

Two reports included pediatric patients developing FAT early after cardiac surgery [16, 20]. The mechanism is unclear, though local atrial trauma inducing abnormal automaticity is suspected, as structural or macro‐reentrant causes are less likely immediately postoperatively. In one study, about half of early postoperative FAT cases did not respond to ivabradine, suggesting non‐HCN mediated mechanisms [16]. Bradycardia was observed in some patients, highlighting the need for careful monitoring. In one report, ivabradine was gradually tapered and discontinued after achieving hemodynamic stability, sinus rhythm, and target heart rate [20].

### 4.4 Ivabradine‐Sensitive Atrial Tachycardia (ISAT) Predictors

In prospective cohorts, the prevalence and characteristics of ivabradine‐sensitive atrial tachycardia (ISAT) vary. Banavalikar et al. reported an ISAT rate of 64% (18 of 28 patients), noting that these tachycardias more often originated from the atrial appendages [19]. In contrast, Xu et al. observed a 50% ivabradine success rate (6 of 12 patients); while no clear predictors of ISAT versus non‐ISAT emerged, most ISATs (83.3%) arose from the right atrium [18]. Exceptions to ivabradine responsiveness highlight the role of the underlying mechanism. Janson et al. described a post‐transplant FAT case in which ivabradine failed to control rate or rhythm, likely reflecting the non‐HCN mediated nature of FAT in pediatric heart transplant recipients [31]. Similarly, Tolani et al. reported a 20% failure rate in their cohort of 15 congenital heart disease patients, all occurring in postoperative FAT, suggesting that micro‐reentry and triggered activity may contribute to ivabradine insensitivity, since postoperative surgical scarring frequently creates substrates for these non‐automatic mechanisms [16].

**FIGURE 3:**
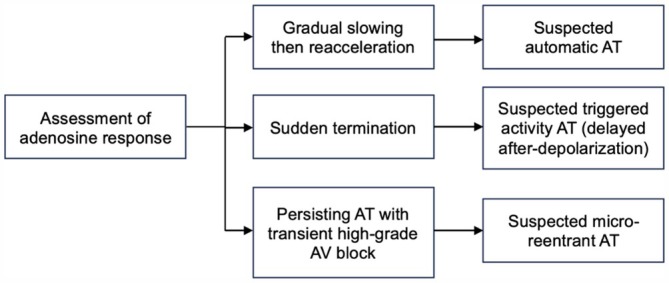
Algorithm to identify FAT underlying mechanism by assessment of adenosine response: Gradual slowing then reacceleration as the primary diagnostic indicator for initiating ivabradine therapy [48].

Identifying ISAT in clinical practice relies on mechanistic clues. Adenosine administration and careful telemetry review can help determine whether automaticity underlies the tachycardia (Figure 3). According to the 2019 ESC Guideline for SVT, features that suggest increased automaticity include: (a) incessant FAT episodes, (b) poor response to conventional antiarrhythmic therapy or electrical cardioversion, and (c) a “warm‐up” and “cool‐down” pattern of onset and termination [48]. The warm‐up and cool‐down pattern remains one of the most reliable predictors of ISAT, as it favors an automatic mechanism. In 2011, Toyohara et al. performed electrophysiological studies (EPS) in 35 pediatric patients with FAT and observed the warm‐up and cool‐down phenomenon in all cases [51]. Clinically, a gradual increase in heart rate before tachycardia onset and a progressive decrease in heart rate before termination on bedside monitoring support an automatic mechanism of FAT.

### 4.5 Reversal of Tachycardia‐Induced Cardiomyopathy

Tachycardia‐induced cardiomyopathy (TIC) is a frequent and concerning consequence of incessant focal atrial tachycardia [52]. Across the studies we reviewed, TIC was reported in roughly one‐third of cohort patients (13 of 40; 32.5%) [18, 19] and in 60% of case report patients (9 of 15) [16, 17, 29, 30, 31, 32, 33, 34], emphasizing its strong association with prolonged automatic FAT. The mechanism involves prolonged rapid atrial rates increasing hemodynamic load on the left ventricle, which can lead to systolic dysfunction.

Ivabradine appears particularly effective in functionally reversing TIC when FAT is automatic and HCN‐dependent. In the available studies that assessed left ventricular function post‐treatment, nearly all patients demonstrated improvement. For example, Ahmed et al. and Tasci et al. reported normalization of LV function in adult and pediatric patients with severe TIC after initiation of ivabradine, sometimes in combination with beta‐blockers or amiodarone [22, 26]. Similarly, Al Musaad and Bohora observed marked LVEF recovery after successful rate control with ivabradine, even in patients previously refractory to conventional therapy [33, 34]. Importantly, FAT termination could occur rapidly, as time to sinus rhythm ranged from as little as 45 min in critically ill infants to several hours in adults, illustrating the potency of targeted HCN inhibition [27]. Although dosing strategies varied across studies, ivabradine consistently achieved effective rate reduction and reversal of tachycardia‐induced cardiomyopathy. Its non‐invasive nature and favorable tolerability make it a practical option across a wide range of patients. Early recognition of TIC and timely ivabradine use in HCN‐dependent FAT may help prevent or reverse left ventricular dysfunction.

The improvement in LV function may also be related to a key feature of ivabradine that distinguishes it from other antiarrhythmic agents, which is the absence of negative inotropic effects. Elzeneini et al. provided a comprehensive review on the use of ivabradine in combination with intravenous inotropic therapy in advanced heart failure, demonstrating how this approach can optimize cardiac output [53]. By selectively inhibiting the I_f_ current through binding to the HCN4 channel, ivabradine produces pure negative chronotropy without compromising myocardial contractility [53]. This property is particularly beneficial in patients with tachycardia and impaired LV contractility, as conventional rate‐control strategies, such as beta‐blockers, are often limited by further systolic depression, and inotropic support, when required, could lead to tachycardia exacerbation. In this context, ivabradine fills an important therapeutic gap as a pure negative chronotropic agent in acute heart failure with impaired contractility, offering an alternative to beta‐blockers while allowing safe use alongside inotropic therapy [53]. One limitation lies in the lack of an intravenous formulation, which should be a focus of future studies [53].

### 4.6 Ivabradine Adverse Effect, Toxicity, and Contraindications

Ivabradine is generally well tolerated, with a favorable safety profile across the studies included. The most commonly reported adverse effects include atrial fibrillation, sinus node dysfunction, atrioventricular block, and bundle branch block, with occasional reports of blurred vision [29, 53]. Long‐term follow‐up data reinforce its safety: in adults with FAT, five studies monitored patients on ivabradine for durations ranging from 3 months to 3 years, without major complications [17, 19, 21, 29, 33]. Similarly, pediatric studies with follow‐up between 10 weeks and 6 months reported no significant side effects [18, 24, 27, 30, 31, 34]. In one report, a pregnant patient treated from the third trimester through 1 month postpartum experienced no adverse events.

Bradycardia remains the most notable clinical concern. Tolani et al. observed transient bradycardia in 47% of pediatric CHD patients with FAT, particularly in those under 1 year or postoperatively [16]. However, the study also found that withholding one dose or halving the total dose of ivabradine, as performed in 57% of patients who developed bradycardia, successfully resolved the bradycardia, while 29% of cases resolved spontaneously, and 14% resolved after ivabradine was replaced with flecainide, highlighting that halving the total dose may be adequate and should be considered the first management option when bradycardia occurs [16]. Careful monitoring remains important in neonates, particularly those < 1 year old with CHD, as significant bradycardia events are more likely to happen in this population due to their reliance on chronotropic competence [16].

Reports of ivabradine toxicity are rare but informative. Two adult cases involved severe sinus bradycardia and even cardiogenic shock, successfully managed with atropine, isoproterenol, and transvenous pacing [29, 34]. A unique case in a 3‐day‐old neonate with FAT highlights the dose–response relationship: the infant received ten times the planned dose (0.5 mg/kg instead of 0.05 mg/kg), developed sinus bradycardia, yet maintained stable hemodynamics. After stopping ivabradine and amiodarone, the neonate's FAT episodes became fewer, possibly reflecting both the high‐dose suppression of automaticity and immature hepatic metabolism; CYP3A4 activity at 1 month of age is only 30%–40% of adult levels [54]. This case illustrates the delicate balance of efficacy and safety in neonates, as well as the potential for enhanced antiarrhythmic effect at higher doses.

Ivabradine is contraindicated in patients with severe hepatic or renal dysfunction due to its hepatic metabolism and renal excretion [26, 48]. Concomitant use with moderate or strong CYP3A4 inhibitors, such as verapamil or diltiazem, should be avoided, while mild inhibitors like amiodarone can be combined cautiously with close monitoring of hepatic function [36]. In fact, in three included studies, ivabradine was co‐administered with therapeutic doses of amiodarone without adverse reactions, demonstrating that careful combination therapy is feasible in select patients [16, 20, 26]. Overall, ivabradine offers a favorable balance of efficacy and safety, with manageable adverse effects, but requires thoughtful consideration of patient age, comorbidities, and concomitant medications to optimize outcomes.

### 4.7 Limitations of Current Evidence

This review is subject to several limitations. First, because ivabradine was originally developed for heart failure and coronary artery disease, the existing studies evaluating its use for arrhythmia, particularly focal atrial tachycardia, remain limited. Consequently, our review includes mostly case reports and case series, with several unrandomized cohort studies lacking control groups, and due to the nature of these study designs, the level of evidence is low and requires careful interpretation.

Second, the dosing of ivabradine varied across studies due to the absence of standardized regimens for focal atrial tachycardia, which may have influenced the reported outcomes. Third, although clinically difficult to avoid unless specifically designed, most cases involved concomitant use of other antiarrhythmic agents prior to ivabradine, which may have introduced bias. Fourth, follow‐up duration was limited, understandably so, given that most included reports were single cases or small series, and the available cohort studies had relatively short follow‐up periods. This is important not only when interpreting treatment efficacy but also when assessing the safety profile of ivabradine.

Despite these limitations, we believe this review represents the best current synthesis of the available evidence regarding ivabradine for focal atrial tachycardia. Future studies, preferably randomized and conducted in controlled populations, are needed to better understand the efficacy and safety of ivabradine for focal atrial tachycardia and for arrhythmias in general.

## 5 Conclusion

Our review found that recognizing an automaticity‐driven FAT is key to considering ivabradine, especially when FAT persists despite cardioversion or standard therapy. Evidence from small cohorts and case reports suggests promising outcomes and a good safety profile, but larger and more robust studies are needed. Data on its use in critically ill patients remain very limited. Well‐designed randomized prospective studies with standardized dosing and longer follow‐up are required to clarify its efficacy and safety.

## Funding

The authors have nothing to report.

## Ethics Statement

The authors have nothing to report.

## Consent

The authors have nothing to report.

## Conflicts of Interest

The authors declare no conflicts of interest.

## Supporting information


**Table S1:** MeSH terms of searching strategy.

## Data Availability

Data sharing not applicable to this article as no datasets were generated or analysed during the current study.
