# Supplementary material for: The Evolving Role of Ivabradine for Focal Atrial Tachycardia: A Systematic Review
Source: J Arrhythm. 2026 Mar 10;42(2):e70312. doi: 10.1002/joa3.70312 (PMC12972838; doi:10.1002/joa3.70312)
Supplement: Supplementary file 1 — Table S1: MeSH terms of searching strategy. [file JOA3-42-e70312-s001.docx]

| Database | Keywords |
| --- | --- |
| PubMed | (ivabradine[Mesh] OR ivabradine)  AND  ("Atrial Tachycardia"[Mesh] OR "atrial tachyarrhythmia*" OR “Focal Atrial Tachycardia”) |
| Scopus | ( ivabradine )  AND  ( "atrial tachycardia" OR “Focal Atrial Tachycardia”) |
| Science Direct | ivabradine  AND  ("atrial tachycardia" OR “Focal Atrial Tachycardia”) |

Supplementary Table 1. MeSH terms of searching strategy.
